# Cerebellar hematoma in severe hemophilia with inhibitor on emicizumab prophylaxis: a case report

**DOI:** 10.1186/s13256-023-03783-7

**Published:** 2023-02-23

**Authors:** Sami Albattat, Abbas Alabdultaif, Fatimah Albattat, Batla Albattat

**Affiliations:** 1 Pediatric Department, Maternity and Children’s Hospital, Alhassa, Ministry of Health, Najran, Saudi Arabia; 2 King Faisal University, Alhassa, Saudi Arabia

**Keywords:** Emicizumab, Hemophilia A, Cerebral hemorrhage

## Abstract

**Background:**

Emicizumab is a novel prophylactic medication used to treat patients with hemophilia A. It is indicated to minimize the frequency of bleeding episodes and the severity of serious bleeding in patients with hemophilia A utilizing inhibitors. However, some cases of bleeding episodes have been reported, and more data are needed regarding their management and expected outcomes.

**Case presentation:**

We report a case of a 4-year-old Saudi Arabian boy with severe hemophilia A who presented with a post-traumatic cerebral hemorrhage. The patient, with high titer inhibitors, was on emicizumab prophylaxis therapy. On hospital admission, he received tranexamic acid (10 mg intravenously, every 6 hours), and recombinant activated factor VII 120 µg/kg every 2 hours for 2 days then every 4 hours for 4 days. On follow-up, the patient showed no signs of neurological deficit. There was no need for emergency neurosurgical intervention since the bleeding had been controlled throughout the first 2 days. There were no recorded thrombotic sequelae or neurological complications, with complete resolution within 10 days.

**Conclusions:**

This case implies that low-dose recombinant activated factor VII might be used safely and effectively with patients with hemophilia A on emicizumab prophylaxis, to reduce the risk of cerebral hemorrhage or another episode of serious bleeding along with its long-term complications.

## Background

Hemophilia is a rare congenital, recessive X-linked disorder resulting from coagulation factor deficiency. Hemophilia A, the most common type, is characterized by a deficiency in the blood clotting factor VIII (FVIII). According to the World Federation of Hemophilia report, there are 1067 patients with hemophilia in Saudi Arabia; 82% of them have hemophilia A [1, 2]. The disease severity depends on the level of depletion of factor VIII [3]. Patients with severe hemophilia A may bleed excessively after injuries, and have spontaneous bleeding episodes. Recurrent bleeding can cause long-term and life-threatening complications, such as hemarthrosis and intracranial bleeding [4].

Challenges in hemophilia treatment include the development of inhibitors or neutralizing antibodies to infused clotting factors [5]. High inhibitor levels need nontraditional approaches for treating bleeding episodes, such as bypassing agents [6]. Activated prothrombin complex concentrates and recombinant activated coagulation factor VII (rFVIIa) are bypassing agents that might be utilized to prevent or manage hemophilia-induced bleeding [7]. For instance, emicizumab is the first humanized bispecific antibody approved for treating patients with hemophilia A. It replaces the hemostatic effects of FVIII products by binding FIXa and FX together [8]. Prophylactic emicizumab administration successfully reduced the frequency of bleeding episodes in patients with severe hemophilia A who have developed inhibitors, as revealed in the HAVEN 1 and 2 studies [9]. However, the impact of emicizumab on reducing bleeding severity remains unclear. This study aimed to describe the case of a 4-year-old boy with severe hemophilia A and a high-titer inhibitor on emicizumab prophylaxis, who developed post-traumatic cerebral hemorrhage.

## Case presentation

A 4-year-old boy from Saudi Arabia was diagnosed with hemophilia A at the age of 15 months, with an initial presentation of gum bleeding following direct trauma. First-degree consanguinity was reported; however, there was no family history of bleeding disorders. Since diagnosis, he has experienced more than three episodes of bleeding annually. He previously received multiple doses of standard recombinant FXIII, and high titer inhibitors were detected, reaching 167 BU/mL. Due to the parent’s poor compliance, prophylaxis treatment was delayed, and he frequently missed doses of recombinant factor VIII Fc fusion protein. Furthermore, immunological tolerance induction (ITI) was not initiated, as it needed frequent injections and his parents refused the central line procedure, or to administer injections independently. Accordingly, he received a subcutaneous emicizumab loading dose of 1.5 mg/kg for 4 weeks, followed by a maintenance dose of 6 mg/kg once every 4 weeks.

**Fig. 1:**
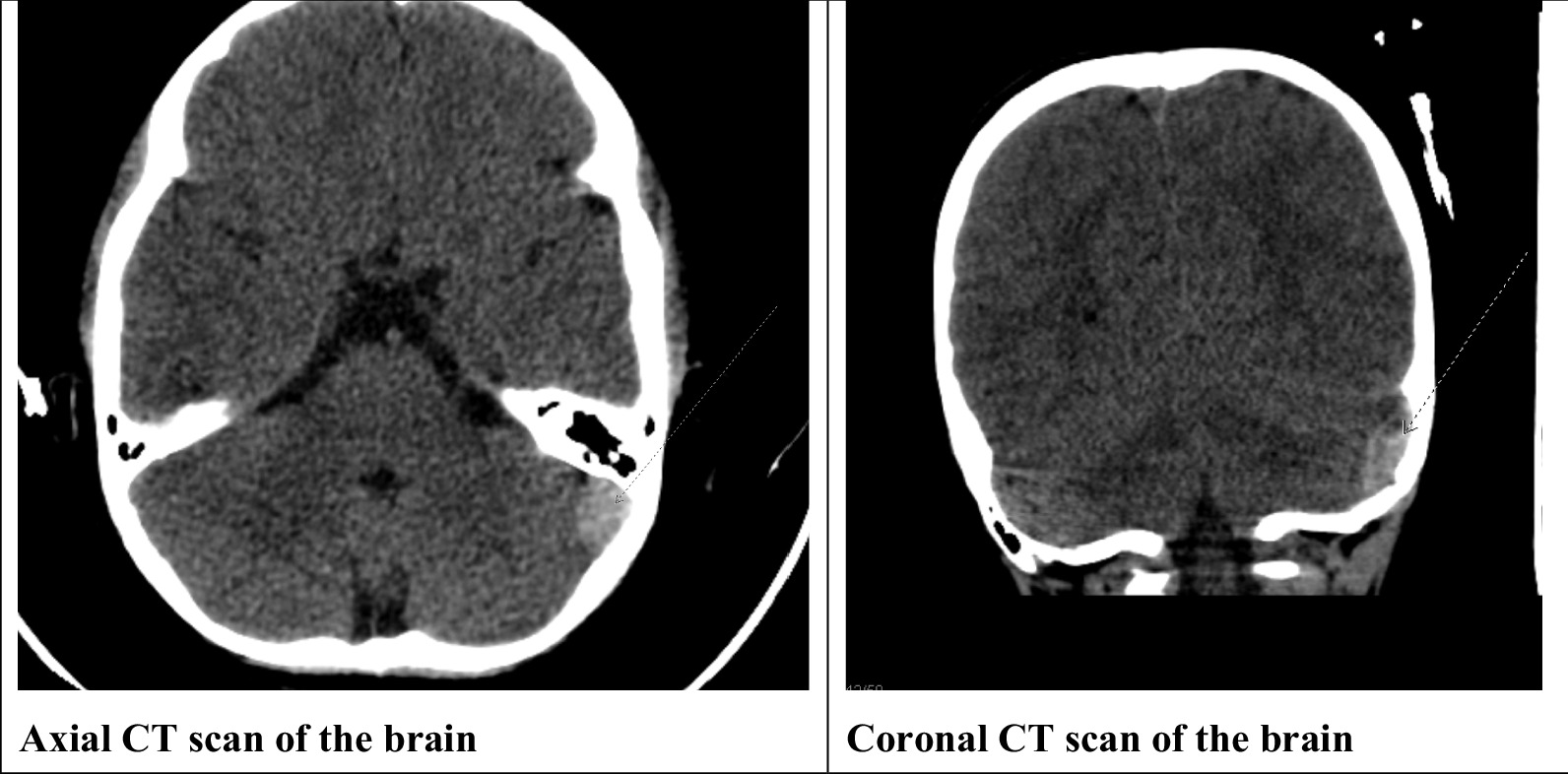
A plain axial CT scan of the brain showing mild interval regression of the left-sided posterior fossa/cerebellar epidural hematoma

The patient arrived at the emergency department 2 hours after experiencing head trauma from falling on wet ground. He suffered from post-traumatic headaches and acute vomiting. On arriving at the emergency department of Maternity and Children’s Hospital, Alhassa, he was conscious, attentive, agitated, and with stable vital signs, including heart rate, blood pressure, temperature, and respiratory rate. Laboratory investigations showed that his hemoglobin was 9.7 g/dL, hematocrit was 30.7%, mean corpuscular volume was 54 fL, reticulocyte count was 1.1%, and white blood cell count was 9.86 × 10^9^/L. On clinical examination, there were no neurological deficiencies regarding reflexes, sensation, mental, cognitive, visual, speech, gait, or motor functions. A computerized tomography (CT) scan of the brain was requested as a part of the routine evaluation and revealed a left-sided posterior fossa cerebellar epidural hematoma (Fig. 1). The patient was admitted and closely monitored in the intensive care unit and received 24 doses of rFVII (120 µg/kg) for the first 2 days, then six doses daily for 4 days, with tranexamic acid (10 mg/kg) given every 6 hours. Brain CT scans were repeated after 2 days, revealing bleeding cessation. Accordingly, no interventional neurosurgical procedures were recommended. The patient showed stable vital signs and normal neurological examination throughout his admission. The patient was discharged after 10 days, in good health, with no neurological complications. One week after discharge, the patient was followed up by the hospital’s neurosurgeon for reassurance.

## Discussion and conclusions

Patients with severe hemophilia A, characterized by plasma FVIII clotting activity of less than 1% of the normal level, are at high risk for experiencing spontaneous and severe bleeding [10]. Approximately 25–40% of patients with severe hemophilia A acquire neutralizing antibodies (inhibitors) against FVIII, making them resistant to FVIII replacement therapy [5]. Notably, young patients usually develop inhibitors during the first 20–40 exposure days to FVIII replacement therapy [11]. Moreover, the only technique that has been proven to eradicate inhibitors is ITI, which is a difficult parental decision since it entails regularly injecting FVIII concentrates and invasive catheterization [12].

The prevalence of intracranial hemorrhage (ICH) was estimated to range from 3% to 12% among patients with congenital hemophilia. Approximately 5–10% of people with severe hemophilia will have more than one ICH in their lifetime [13]. Children with hemophilia are more likely to suffer head traumas, and factor replacement treatment, which is necessary to treat ICH, is associated with a higher risk of inhibitor development. It was reported that patients with hemophilia, who have inhibitors, should receive bypassing medicines as soon as symptoms appear and should keep receiving them after an ICH event [14]. Due to the high hazards and high mortality rate associated with ICH, it should be treated presumptively before any examination because it is a true medical emergency [15].

Subcutaneous administration of emicizumab provides high bioavailability and sustained therapeutic trough plasma concentrations. It has a half-life of approximately 30 days, enabling treatment once weekly [16]. However, emicizumab provides sufficient hemostasis to prevent severe spontaneous bleeding episodes in most patients with hemophilia. It has been thought that emicizumab does not preclude significant trauma-induced bleeding or spontaneous bleeding in patients with inhibitors; therefore, patients receiving emicizumab prophylaxis should be considered at high risk of acute bleeding. Even after emicizumab has established baseline hemostasis, acute bleeding events may still necessitate intensive management [17].

Many studies have highlighted the promising results of adding emicizumab to the bypassing agent treatment. A 10-year-old boy from Chile was diagnosed with severe hemophilia A with inhibitors. Due to frequent episodes of breakthrough bleeding, his normal daily activities were highly restricted. Prior to receiving therapy with emicizumab, he was receiving episodic recombinant FVIIa treatment and had experienced 18 episodes of bleeding. After a year of emicizumab prophylaxis, the patient had only one bleeding episode (a reduction of 94.4%), better pain control (5 points on the visual analog scale), a reduction in the Hemophilia Joint Health Score (from 39 to 19), an increase in quality of life (QoL) perception of 86% on the standardized Haemo-QoL-kids, and a reduction in treatment costs of 70% compared with the costs of episodic treatment with recombinant FVIIa [18]. Eleven children with severe hemophilia A and inhibitors were included in the study of Barg *et al*. None of the patients had hemarthrosis or any other kind of spontaneous bleeding throughout the follow-up period. Emicizumab prophylaxis effectively prevented bleeding for 63.63% of the patients who required no further therapies. Only 4 out of 11 individuals received recombinant-activated FVII occasionally [19]. These findings support the results of this case, in terms of the safety and efficacy of the combination of emicizumab and recombinant treatment.

In this case, we believe that emicizumab contributed significantly to minimizing the bleeding after serious head trauma. Emicizumab can function safely and efficiently regardless of the presence of the FVIII inhibitors titer, and it does not require activation by thrombin. Thus, we could initially manage our case by using rFVII and tranexamic acid as antifibrinolytic agents. Moreover, we advocate using emicizumab as a prophylaxis agent, especially with pediatric patients.

Pediatric patients with hemophilia A can develop a high titer of inhibitors against the replacement factor. They are susceptible to head trauma, which increases the risk of significant bleeding, including cerebral hemorrhage, a lethal disease with high mortality. Emicizumab as a prophylaxis drug may reduce the frequency of bleeding events, even with the inhibitors’ presence. The clinical outcome of this patient showed that rFVIIa, in combination with tranexamic acid and emicizumab, is safe and effective. Further well-designed studies are required to prove the efficacy of emicizumab in patients with severe hemophilia with high titer of inhibitors.

## Data Availability

The data and materials supporting the findings of this study are available on request from the corresponding author.

## Abbreviations

ICH: Intracranial hemorrhage
F: Factor
rFVIIa: Recombinant activated factor VII
CT: Computerized tomography

## References

[1] World federation of hemophilia. Annual Global Survey 2020. 2020;96. https://wfh.org/data-collection/. 10.1111/hae.14012 32497379

[2] Umar D, Baroudi K, Kaul R, Shastry S (2014). Hemophilia A: dental considerations and management. J Int Soc Prevent Communit Dent.

[3] Castaman G, Matino D (2019). Hemophilia A and B: molecular and clinical similarities and differences. Haematologica.

[4] Rodríguez-Merchán EC, De Pablo-Moreno JA, Liras A (2021). Gene therapy in hemophilia: recent advances. IJMS.

[5] Kruse-Jarres R, Kempton CL, Baudo F, Collins PW, Knoebl P, Leissinger CA (2017). Acquired hemophilia A: updated review of evidence and treatment guidance. Am J Hematol..

[6] Merlin S, Follenzi A (2020). Escape or fight: inhibitors in hemophilia A. Front Immunol.

[7] Giansily-Blaizot M, Schved JF (2017). Recombinant human factor VIIa (rFVIIa) in hemophilia: mode of action and evidence to date. Ther Adv Hematol.

[8] Gelbenegger G, Schoergenhofer C, Knoebl P, Jilma B (2020). Bridging the missing link with emicizumab: a bispecific antibody for treatment of hemophilia A. Thromb Haemost.

[9] Mahlangu J, Oldenburg J, Paz-Priel I, Negrier C, Niggli M, Mancuso ME (2018). Emicizumab prophylaxis in patients who have hemophilia A without inhibitors. N Engl J Med.

[10] Butenas S, Parhami-Seren B, Undas A, Fass DN, Mann KG (2010). The “normal” factor VIII concentration in plasma. Thromb Res.

[11] Abdi A, Eckhardt CL, van Velzen AS, Vuong C, Coppens M, Castaman G (2021). Treatment-related risk factors for inhibitor development in non-severe hemophilia A after 50 cumulative exposure days: a case–control study. J Thromb Haemost.

[12] Lacroix-Desmazes S, Voorberg J, Lillicrap D, Scott DW, Pratt KP (2020). Tolerating factor VIII: recent progress. Front Immunol.

[13] Singleton TC, Keane M (2012). Diagnostic and therapeutic challenges of intracranial hemorrhage in neonates with congenital hemophilia: a case report and review. Ochsner J.

[14] Zanon E, Pasca S (2019). Intracranial haemorrhage in children and adults with haemophilia A and B: a literature review of the last 20 years. Blood Transfus.

[15] Gulati D, Dua D, Torbey MT. Hemostasis in Intracranial Hemorrhage. Front Neurol. 2017;8. 10.3389/fneur.2017.00080/full. 10.3389/fneur.2017.00080 PMC5351795 28360881

[16] Schmitt C, Adamkewicz JI, Xu J, Petry C, Catalani O, Young G (2021). Pharmacokinetics and pharmacodynamics of emicizumab in persons with hemophilia A with factor VIII inhibitors: HAVEN 1 study. Thromb Haemost.

[17] Zimowski KL, Batsuli GM, Bryant P, McDaniel J, Tickle K, Meeks SL (2019). Severe bleeding events in hemophilia Α patients receiving emicizumab prophylaxis. Blood.

[18] Abarca-Villaseca V, Soto-Arellano V. Breakthrough bleeding episodes at minimum and improvement in quality of life in a child with severe hemophilia A with inhibitors treated with emicizumab: a case report from Chile. Am J Case Rep. 2021;22. Available from: https://www.amjcaserep.com/abstract/index/idArt/929598. 10.12659/AJCR.929598 PMC8077390 33883542

[19] Barg AA, Avishai E, Budnik I, Levy‐Mendelovich S, Barazani TB, Kenet G, *et al*. Emicizumab prophylaxis among infants and toddlers with severe hemophilia A and inhibitors—a single‐center cohort. Pediatr Blood Cancer. 2019;66(11). 10.1002/pbc.27886. 10.1002/pbc.27886 31348595

